# Development and validation of a rapidly deployable CT-guided stereotactic system for external ventricular drainage: preclinical study

**DOI:** 10.1038/s41598-021-97080-2

**Published:** 2021-09-01

**Authors:** Abhijeet S. Barath, Aaron E. Rusheen, Juan M. Rojas Cabrera, Hojin Shin, Charles D. Blaha, Kevin E. Bennet, Stephan J. Goerss, Kendall H. Lee, Yoonbae Oh

**Affiliations:** 1 Department of Neurologic Surgery, Mayo Clinic, Rochester, MN 55905 USA; 2 Mayo Clinic Graduate School of Biomedical Sciences, Mayo Clinic, Rochester, MN 55905 USA; 3 Medical Scientist Training Program, Mayo Clinic, Rochester, MN 55905 USA; 4 Division of Engineering, Mayo Clinic, Rochester, MN 55905 USA; 5 Department of Biomedical Engineering, Mayo Clinic, Rochester, MN 55905 USA; 6 NaviNetics Inc., Rochester, MN 55905 USA

**Keywords:** Brain injuries, Hydrocephalus

## Abstract

External ventricular drainage (EVD) is an emergency neurosurgical procedure to decrease intracranial pressure through a catheter mediated drainage of cerebrospinal fluid. Most EVD catheters are placed using free hands without direct visualization of the target and catheter trajectory, leading to a high rate of complications- hemorrhage, brain injury and suboptimal catheter placement. Use of stereotactic systems can prevent these complications. However, they have found limited application for this procedure due to their long set-up time and expensive hardware. Therefore, we have developed and pre-clinically validated a novel 3D printed stereotactic system for rapid and accurate implantation of EVD catheters. Its mechanical and imaging accuracies were found to be at par with clinical stereotactic systems. Preclinical trial in human cadaver specimens revealed improved targeting accuracy achieved within an acceptable time frame compared to the free hand technique. CT angiography emulated using cadaver specimen with radio-opaque vascular contrast showed vessel free catheter trajectory. This could potentially translate to reduced hemorrhage rate. Thus, our 3D printed stereotactic system offers the potential to improve the accuracy and safety of EVD catheter placement for patients without significantly increasing the procedure time.

## Introduction

External ventricular drainage (EVD) is an emergency, life-saving procedure for patients presenting with increased intracranial pressure (ICP), not responding to medical management. It involves implantation of a catheter in the ventricles of the brain to drain cerebrospinal fluid (CSF) and reduce ICP. Currently, most EVD catheters worldwide are implanted using the free hand technique. In this technique the surgeon uses standard external anatomical landmarks to define a point of entry on the skull and blindly guides the catheter towards the target (foramen of Monroe, FOM) using their free hand^1^. Thus, this technique does not directly visualize the target, or the blood vessels along the catheter trajectory, and does not take into account deviations from normal anatomy. This contributes to a high rate of mechanical complications including- hemorrhage (2.8–28%)^1–5^, cerebrovascular injury (2.75%)^6^, and suboptimal catheter placement (13–20%)^1,2,7^. Most hemorrhages are clinically silent with symptomatic hemorrhages resulting in new neurologic deficits in 2–2.5% patients^4,8^. Between 3.2 and 11% of catheters need repositioning or replacement due to suboptimal placement into the thalamus, internal capsule or other structures^1–3,9^. In addition, several studies have reported around 2 attempts^5,10–14^ per successful catheterization, with up to 20 witnessed maximum attempts^11^, reported in an anonymous survey. Although EVD associated hemorrhage is multifactorial and involves factors such as platelet count at admission^5^, the number of EVD placement attempts has been identified as an important contributor to increased rates of hemorrhage, higher bleed volumes, and morbidity^4,5^. While some of these figures seem deceptively small, they translate into a very high morbidity given that nearly 25,000 procedures are performed annually in the US alone^15^.

Image-guided placement of EVD catheters with and without devices such as the Ghajar guide have been previously trialed as an alternative to the freehand technique^12–14,16,17^. Although these have improved the accuracy of catheter placement, they continue to experience significant limitations. These include an increased dose of radiation from repeated CT scans, need for specialized ultrasound probes or bulky neuronavigation hardware, limited trajectories available for selection, and increased total procedural time^14,17^. These limitations with current devices prevent widespread adoption of stereotaxy for EVD catheter placement. Therefore, we need a method that incorporates individual patient anatomy, allows precise control of catheter trajectory, preserves current surgical workflow, and can be conducted in an expedited timeframe.

We have developed and pre-clinically validated a novel 3D printed stereotactic system for EVD catheter implantation which meets the above-described criteria. The mechanics of this system were designed to facilitate a fast and user-friendly surgical workflow through use of a compact skull-mounted EVD key and a stereotactic targeting device that can be handled by a single operator. We have achieved a clinically desirable accuracy (< 5 mm) and a robust safety profile through a rigorous three level testing scheme which includes—mechanical and imaging accuracy tests and a human cadaveric preclinical study.

## Results

### The surgical system

**Figure 1:**
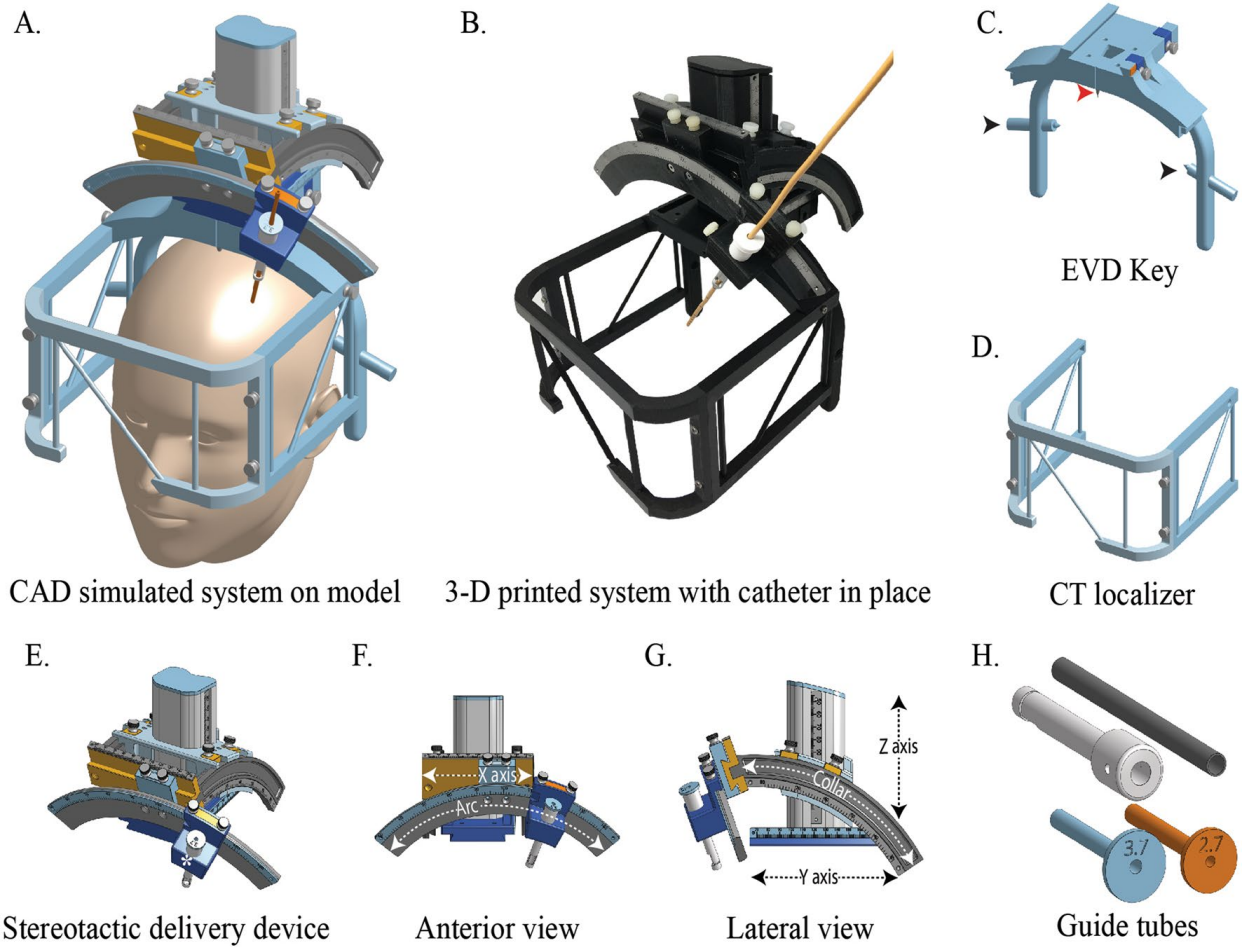
Stereotactic system for external ventricular drainage (EVD) catheter placement. (**A**) CAD model of the complete system mounted on a head with the EVD catheter in place (dark orange); (**B**) 3D printed system with EVD catheter in situ; (**C**) skull-mounting device- the EVD key with 4 fixation points -two side screws labeled with black arrowheads, a midline anterior pin labeled with a red arrowhead and a midline posterior pin (not seen); (**D**) compact N Bar CT localizer. (**E–G**) Stereotactic targeting device with 3 linear degrees of freedom along, X, Y and Z direction and two angular degrees of freedom- arc and collar angles, respectively. The arc carrier (labeled with a white asterisk in E) serves as the platform for mounting and un-mounting the guide tubes. (**H**) Outer guide tube (grey), drill guide (black) and reducing tubes for different catheter sizes (blue and orange).

The three components of our stereotactic system- base frame, localizer and targeting device were designed using a computer aided software and then 3D printed. The EVD key, functional equivalent of stereotactic base-frame in our system could be attached to the head by a single operator using a clamp mechanism tightened with adjustable side screws (Figs. 1A, C, 3B). Two midline pins provided additional points of fixation besides the side screws (Fig. 1C). The EVD key provides a stable frame of reference with respect to the skull and serves as a platform on which subsequent mechanisms can be mounted.

A CT localizer is used with the key for image registration and surgical planning. N-bar design was used for our compact CT localizer. It consisted of three plates: two on the side and one anterior (Fig. 1A, B, D). Each of the plates had two 90 mm tall parallel vertical rods, separated by 120 mm, and connected by a diagonal rod at 45° from the vertical (Fig. 1D). The anterior plate was placed at 110.45 mm from the center of the side plates. The localizer was designed to have an open top for ready access to the surgical site (Figs. 1B, 3F–H). The dimensions of the localizer were chosen for compatibility with commonly available stereotactic surgical software.

Stereotactic targeting device is used to deliver the catheter to a desired target along a suitable trajectory. Our targeting device was based on the center-of-arc principle with a radius of 150 mm and five degrees of freedom- three linear (X, Y, and Z) and two angular (arc and collar, Fig. 1E–G)^18^. This design allows a target within the work-space to be approached from multitude of directions limited only by the range of arc and collar angles. The work-envelope of this targeting device, i.e. the 3D volume in which targets can be chosen was designed to be 100 × 110 × 80 mm (width x length x height) which adequately cover the common intracranial targets for EVD^18^. The linear scales (X, Y, and Z) could be adjusted to reach any target within this work envelope. The angular scales- arc and collar angles, could also be adjusted in the range of 50° to 140° and 30° to 80°, respectively, for selection of a desired trajectory.

### Mechanical and imaging accuracy

**Figure 2:**
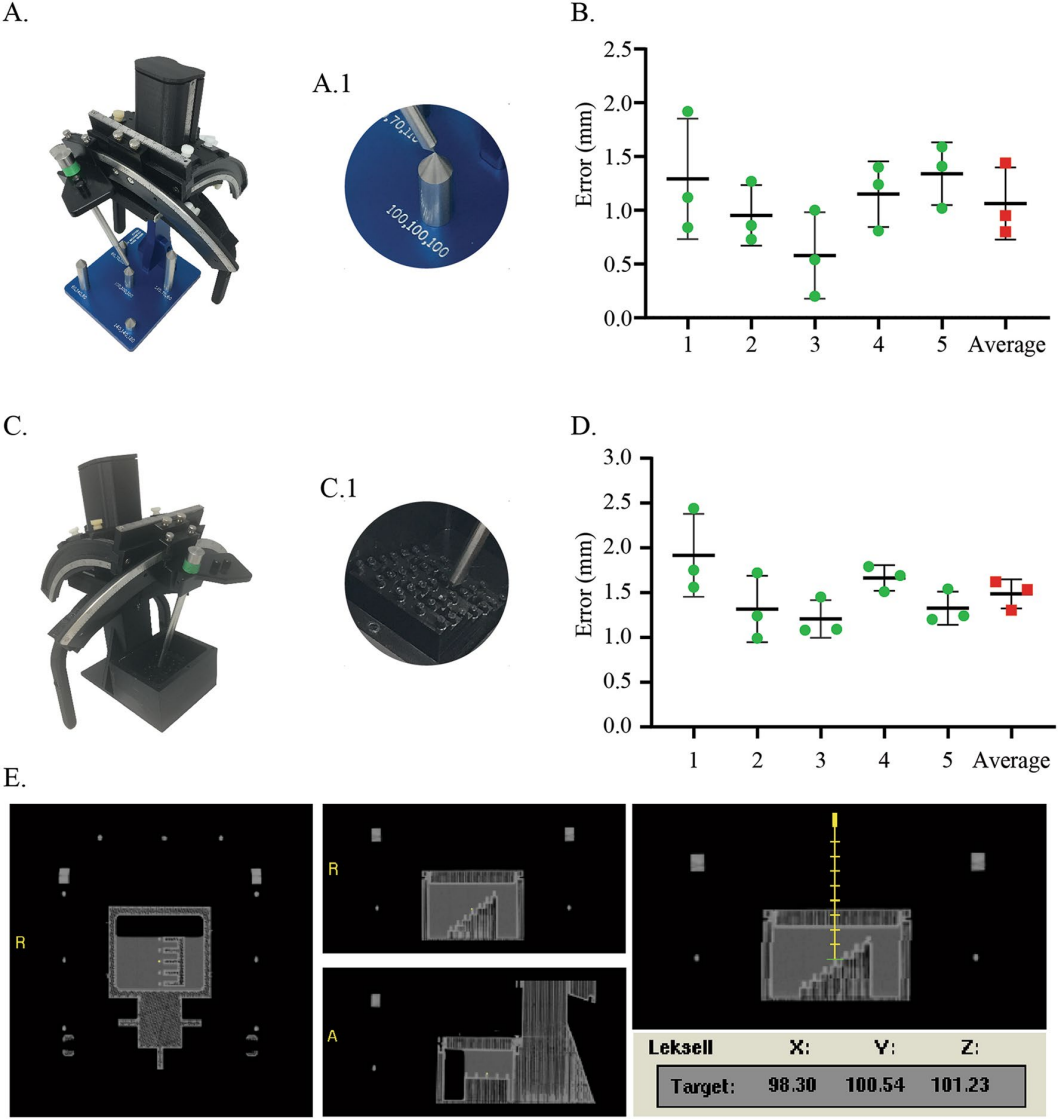
Mechanical and imaging accuracy tests. (**A**) Mechanical accuracy test fixture with zoomed in view of the conical tip being targeted (**A.1**) shown inset. (**B**) Mechanical accuracy test results for all 5 targets (3 independent observers per target). (**C**) Imaging accuracy test fixture with inset (**C.1**) showing all available target points. (**D**) Imaging accuracy test results for 5 target points selected to provide maximal coverage of the work envelope. (**E**) Snapshot of COMPASS surgical planning software showing targeting of a representative point (yellow dot and trajectory line) on imaging test fixture and its coordinate readout.

The mechanical accuracy of the system was found to be 1.1 ± 0.3 mm (Fig. 2B, n = 3 observers). It quantifies the error of the stereotactic delivery device due to its intrinsic mechanics. The imaging error was found to be 1.5 ± 0.3 mm (Fig. 2D, n = 3 observers). It quantifies the combined error from the device mechanics and imaging.

### Cadaveric preclinical study

**Figure 3:**
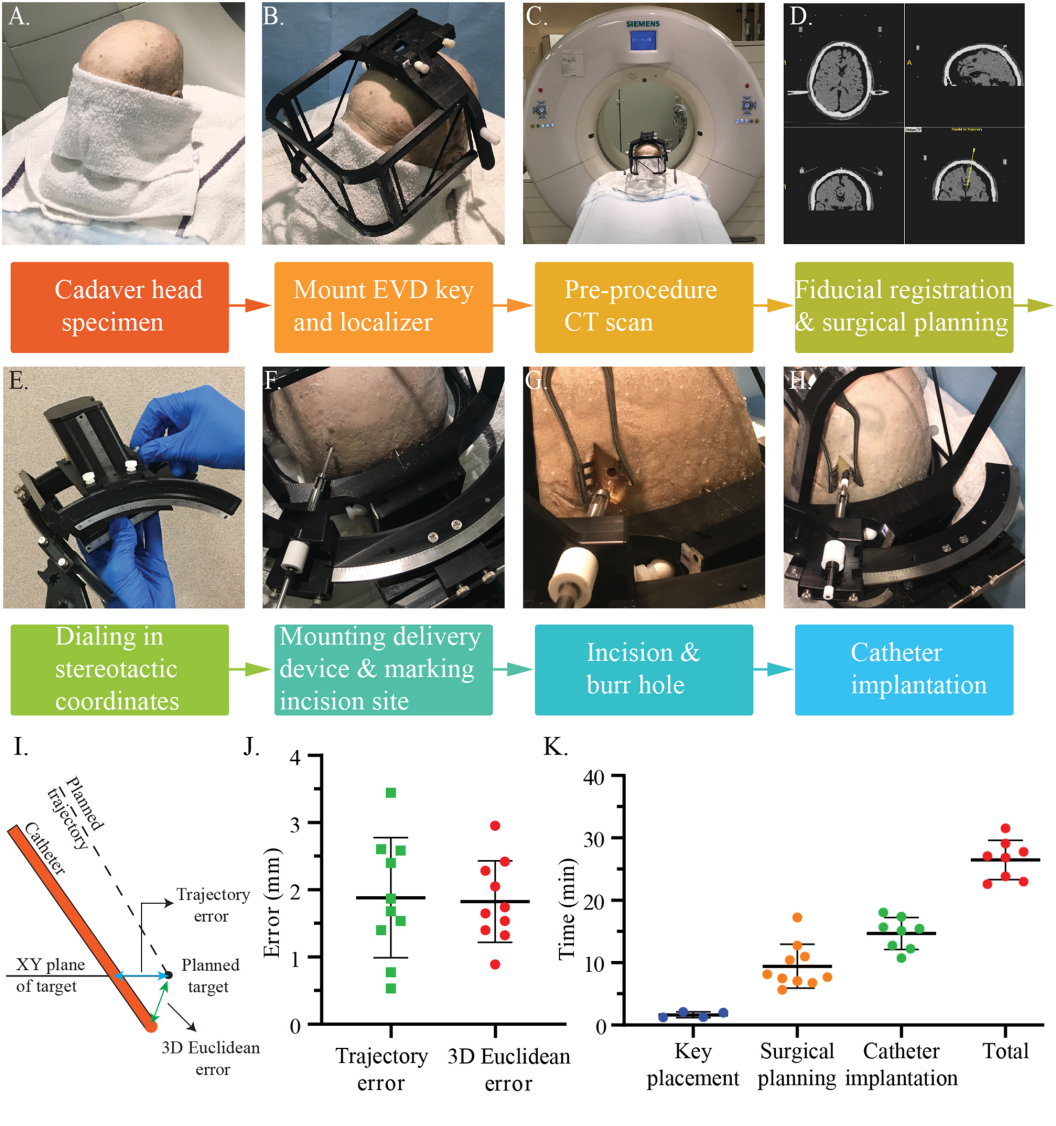
Surgical workflow (**A**–**H**) and results (**I**–**K**) from cadaveric pre-clinical study. (**A**) Specimen positioning for EVD catheter implantation. (**B**) The EVD key and localizer were mounted on the specimen and (**C**) a pre-procedure CT scan was obtained. (**D**) The CT data was transferred to surgical planning software and fiducial registration was performed. Stereotactic coordinates were obtained for a target close to the foramen of Monro and an appropriate trajectory (collar and arc angles) was chosen. (**E**) The stereotactic coordinates, and the arc and collar values obtained in the previous step were dialed into the stereotactic targeting device and the device was mounted on the EVD key. (**F**) A probe was used to mark the site of incision along the trajectory. (**G**) An incision was made at the marked site and a 5 mm burr hole was stereotactically drilled into the bone with the help of a drill guide and a standard #9 drill bit. (**H**) A metal catheter was then implanted into the brain and secured in place. A post-procedure CT scan and target verification was performed thereafter (not shown). (**I**) A schematic explaining the 3D Euclidean error and trajectory error. (**J**) The 3D Euclidean error and targeting error were found to be 1.8 ± 0.6 mm (n = 10) and 1.9 ± 0.8 mm (n = 10) (**K**) Stepwise and total procedure time: key placement (1.6 ± 0.4 min, n = 4), surgical planning (9.4 ± 3.5 min, n = 10), EVD catheter implantation (14.7 ± 2.6 min, n = 8) were completed in total 26.5 ± 3.1 min (n = 8).

**Figure 4:**
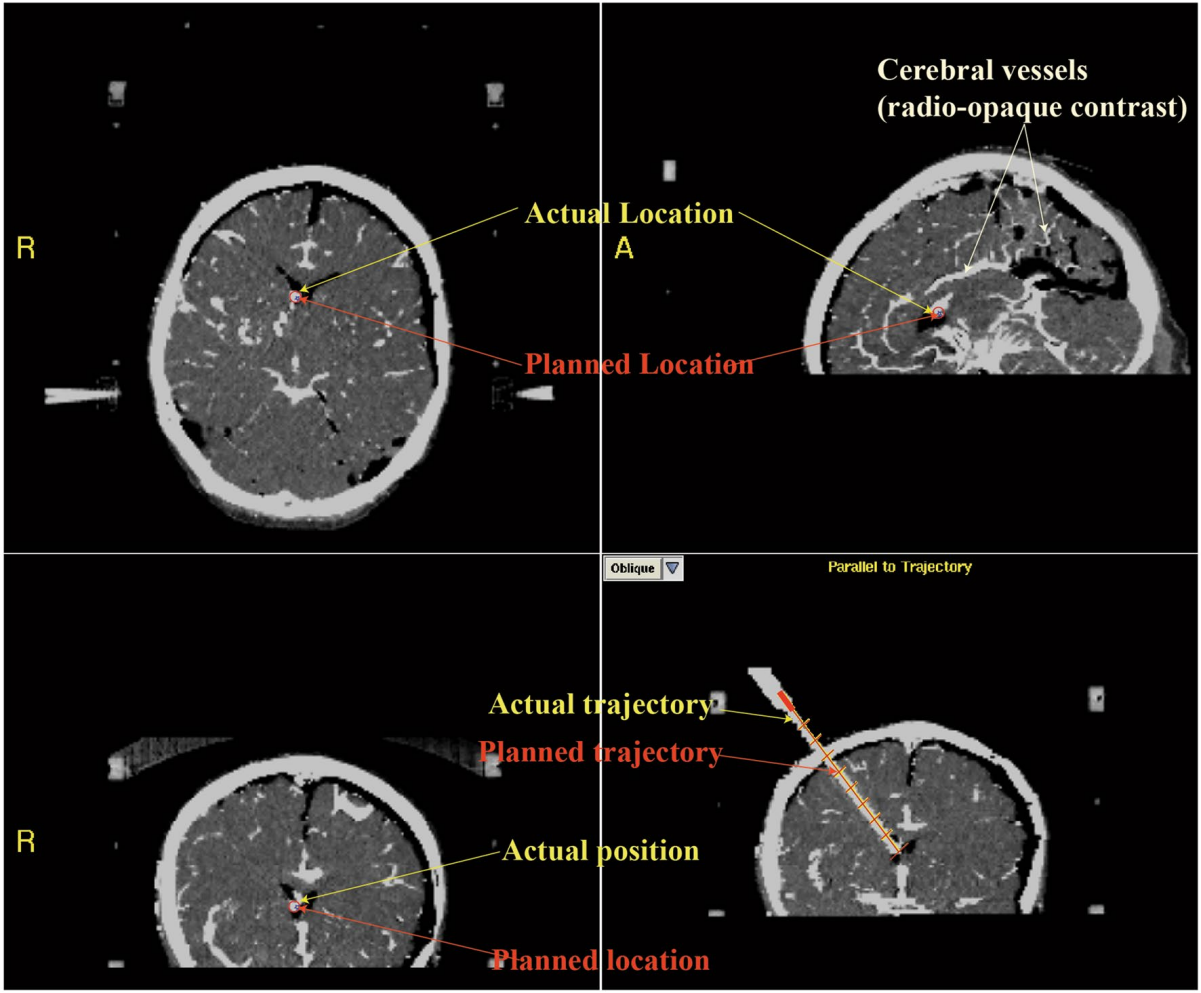
CT images showing robust targeting accuracy in a representative pre-clinical experiment (COMPASS software). The actual location of catheter tip (red circle) is seen to be overlying the planned location (blue dot). The bottom right panel shows the actual catheter trajectory (white glow) with superimposed planned trajectory (red line). Cerebral vasculature was avoided while placing the catheter.

A total of 10 catheter implantations were performed in human cadaver specimens. All catheters were implanted in the frontal horns of lateral ventricles in the first attempt leading to a 100% first pass success rate. The 3D Euclidean error (3D distance) between the software-planned and the frame-targeted coordinates was 1.8 ± 0.6 mm (n = 10, Fig. 3J). We also measured the deviation of the catheter from its trajectory in the XY plane of the target and referred to it as the trajectory error. The trajectory error was found to be 1.9 ± 0.8 mm (n = 10, Fig. 3J). The distance of the margin of catheter tip from the FOM was found to be 3.9 ± 1.6 mm (n = 10). The average time taken for key placement, surgical planning (including fiducial registration), and EVD implantation was 1.6 ± 0.4 min (n = 4), 9.4 ± 3.5 min (n = 10), and 14.7 ± 2.6 min (n = 8) min, respectively (Fig. 3K). Two observations for implantation time were excluded as they employed a pre-existing burr hole. The total time for the procedure excluding the CT scan was 26.5 ± 3.1 min per implant (n = 8, Fig. 3K). Figure 4 shows the targeting accuracy assessment of a representative experiment by superimposing the planned trajectory on the actual trajectory using COMPASS surgical planning software (COMPASS International Innovations, Rochester, MN). The entire procedure was completed on the CT scanner table without transporting the specimen.

The catheter tip was located within ipsilateral frontal horn in all implantations and thus judged Grade I (optimal/adequate) as per Kakarla et al.'s clinical grading system^1^ (supplementary Table S1). For 9 implantations, the catheter tip was located within 5 mm of ipsilateral FOM and judged as ideal based on Fargen et al.’s^4^ grading system (supplementary Table S2). For one implantation, the tip was placed 8 mm from the FOM in the ipsilateral lateral ventricle and judged as acceptable. For experiments emulating surgical planning with CTA, a thorough visual inspection of the post-procedure CT images revealed no direct contact of the catheter with visible vessels (n = 5, see Fig. 4, bottom right panel for representative image).

## Discussion

In this study we have developed and pre-clinically validated a novel stereotactic system for EVD catheter placement. The system was shown to have a robust mechanical, imaging, and overall implantation accuracy with less than 2 mm error. This exceeds current clinical standards (< 5 mm)^4^ as well the accuracy of simpler devices such as the Ghajar guide^13^. The catheter implantations could be performed within 30 min. Additionally, we demonstrated effective utilization of vessel free catheter trajectories. This may translate to reduced incidence of cerebrovascular injury and hemorrhage in patients, and thus lower morbidity. This system’s low-profile design, small footprint, and ability to detach and reattach components on the EVD key without loss of accuracy makes it conducive for use in EVD catheter implantation.

The marked accuracy of this system, demonstrated by catheter tip placement within ~ 4 mm of FOM, illustrates the fundamental advantage of stereotactic guidance. In contrast, the catheter tips are known to be placed around 9.7–16.1 mm away from the FOM with the free hand technique^10,13^. Consequently, suboptimal placement is common and can include eloquent brain areas^1,2,4,19^. This can lead to new neurological complications in patients with existing severe neurological compromise. Higher accuracy is especially desirable in situations where placement of catheters is expected to be difficult, such as with traumatic brain injury, significant cerebral edema, compressed or slit ventricles, and/or significant midline shift^14^. Additionally, stereotactic implantation allows the catheters to be successfully implanted in a single attempt which can lower the cumulative risk of cerebral injury and hemorrhage associated with multiple catheter insertions^4,5^.

The work envelope of this stereotactic system provides an adequate coverage of the common EVD catheter target sites: frontal horns, FOM, and superior part of the third ventricle. The bifrontal diameter of the lateral ventricles has been reported to be between 24 and 42 mm^20,21^ in normal individuals. The mediolateral dimension (X) of the work envelope was chosen to be 100 mm to accommodate enlarged hydrocephalic ventricles and severe midline shifts. The frontal horns are ~ 18 mm high^20^ (measured 3 cm anterior to the coronal suture) and situated ~ 60 mm below the skull surface^22^. The third ventricle extends a few cm below the frontal horns. This is covered well by the Z dimension (80 mm) of the work envelope whose upper limit begins at ~ 22.5 mm below the skull surface. In addition, its large Y dimension (110 mm) provides extensive antero-posterior coverage of the frontal horns. In addition, this wide work envelope could facilitate other procedures like catheter-based hematoma evacuation, quick brain biopsies, and intracranial pressure monitor placement.

Catheter implantations were performed in < 30 min. Increased procedure time is a major deterrent to adoption of image guided and stereotactic surgical systems for EVD placement. In a large survey of ventriculostomy practices, 94% practicing neurosurgeons and 93% residents said they would not use an image-guided system if it added more than 10 min to the procedure time^11^. Time economy was thus a major thrust of our system’s design. The clamp mechanism for securing the EVD key, use of an N-bar fiducial system (which is automatically detected by surgical planning software), the small footprint of the device, and the user-friendly mechanics facilitate an expeditious procedure. On average, EVD key implantation and surgical planning (fiducial registration and target/trajectory selection) were found to take about 1.6 and 9.4 min respectively. This is an improvement over the set up and registration time of electromagnetic neuro-navigation (frameless stereotaxy) which has been found to take approximately 34 min^14^. An advantage of electromagnetic neuronavigation over our system, however, is that surface registration may be performed on a prior volumetric CT scan, making it more suitable for unstable patients^14^. Although the accuracy may be affected if there is ongoing anatomical distortion from the time of scan to the time of the procedure^14^. This disadvantage may be offset by the small footprint of our system which allows catheter implantation to be performed within the CT suite, immediately following the planning scan. Furthermore, Krötz et al. have demonstrated that EVD catheterization in the CT suite can save close to two hours in transportation of the patient to the operating room and back to ICU^12^ without significantly affecting the infection rates^12,19^.

Reduced catheter occlusion rates may be another potential advantage of this system. Pang et al. 1994, and Fargen et al. 2016, have shown that catheters placed just anterior to the FOM and within 5–6 mm of it had reduced rates of obstruction^4,23^. Catheters are commonly blocked by thrombosis, catheter malposition, debris from the choroid plexus, and reactive glial tissue growth in the catheter holes^23–25^. Therefore, it has been proposed that placing the hole-bearing segment of the catheter completely within the ventricle and away from the choroid plexus and injured ependymal/subependymal tissue may reduce occlusion rates^23,24^. This can be achieved by using a stereotactic surgical system via: (a) selection of a target anterior to and within 5 mm of FOM, (b) selecting a trajectory passing through the roof of the lateral ventricle and directed along its coronal obliquity to maximize the length of the catheter within the ventricle, and (c) assured correct placement of the catheter in first attempt.

Increased procedure cost is another major deterrent to adoption of stereotactic systems for EVD. Use of this system would add some overhead cost which remains to be determined. However, the cost may be partially offset by potential savings on catheter revision/replacement. EVD replacement cost has been estimated to be between $1300 and $3200 per replacement at an overall replacement rate of 26%^25^. The revision rate is significantly lower (one third) when catheters are placed with image guidance compared to freehand technique^26^. Furthermore, unlike electromagnetic neuronavigation, this system does not require an operating room for catheter placement. Roach et al., 2019 have shown that EVD catheters implanted outside the operating room could lead to a significant cost saving of USD $1362.24 (£1046) per implantation^19^. Thus, stereotactic placement of EVD catheters with the proposed system may overall be cost-effective.

Our stereotactic surgical system allows the EVD catheter to be inserted through any point on the anterior half of scalp, including the traditional Kocher’s point. It is however important to define a safe entry zone to maximize patient safety. Therefore, based on an extensive survey of neurosurgical literature, we recommend that ventriculostomy burr hole be placed within the following boundaries—(a) medially, 2 cm from the midline to avoid damage to superior sagittal sinus and reduce blood loss^27,28^; (b) posteriorly, 0.5 cm anterior to the coronal suture to avoid damage to motor cortex^29–31^; (c) laterally, the superior temporal line to avoid penetration of temporalis muscle^30,31^ and (d) anteriorly, the hairline to avoid a scar in cosmetically sensitive area of face. These recommendations are also summarized in Figure S2 and Table S3. Selecting an entry point within these boundaries may maximize patient safety.

This study has several limitations. First, the total procedure time noted in this study does not include the surgical preparation and imaging time. Local anesthesia, surgical prepping and draping are estimated to take around 15 min^17^. A CT scan is relatively fast, with < 1 min added for intravascular contrast administration for brain CT angiography^32^. However, for some patients with rapid clinical deterioration, it may not be possible to go back to the CT for a planning scan. Second, low platelet count^5^, prior anticoagulation, and small catheter size^4^ have also been associated with EVD associated hemorrhage and occlusion rates. These cannot be addressed by improving the accuracy of catheter placement. Third, our pre-clinical study protocol was modified from the routine clinical protocol for practical reasons. Formalin hardened cadaver brain caused rumpling of the rubber catheter (Figure S1A) and hardened bone caused the drill bit to slip (Figure S1B and C). Therefore, we used a metal catheter for our experiments and did not perform subcutaneous tunneling. A metal guide tube was introduced to prevent slipping of the drill bit (Fig. 1H). Finally, the efficacy of stereotactic placement in improving patient safety, reducing catheter occlusion, hemorrhage rates, and the overall financial burden can be accurately gauged only with a clinical trial.

## Conclusions and future direction

In conclusion, we have developed a 3D-printed stereotactic system that can be used for an accurate and timely implantation of EVD catheters. This conclusion is supported by the three-level testing including mechanical and imaging accuracy tests as well as cadaveric EVD implantations. The system had an accuracy of less than 2 mm and all cadaver implantations were performed in less than 30 min. In addition, the EVD key (stereotactic base-frame) could be secured and the stereotactic targeting device handled by a single operator. Further, catheter contact with vasculature was avoided with a carefully selected surgical plan. Therefore, this system offers the potential to improve the accuracy and safety of EVD catheter placement for patients using stereotactic guidance, without significantly increasing the procedure time.

In future we aim to further optimize the device design and surgical workflow to facilitate EVD placement in intubated supine patients in intensive care units who often have multiple other lines. This process would be informed by feedback from multiple neurosurgeons performing mock EVD placement with the current device. This latter study would act as a bridge to future feasibility studies of this system.

## Materials and methods

### Development of the Stereotactic System

The stereotactic system was designed on computer-aided design software Onshape (Onshape, Cambridge, MA, Fig. 1A) and 3D printed with Ultimaker S5 3D Printers (Ultimaker, Geldermalsen, Netherlands) using tough polylactic acid material (Fig. 1B). First, we developed a base-frame which can be securely attached to the patient’s head with 4 fixation points—two side screws and two midline pins (Fig. 1C). This base-frame was designed to facilitate quick mounting (compared to classical stereotactic base-frames) and is referred to as the ‘EVD key’. Next, we designed a compact N-bar CT localizer (Fig. 1D). The localizer is co-imaged with the head for pre-operative target planning and post-operative target verification. The surgical planning software utilize this localizer as a frame of reference (also known as fiducials) in the CT scan image volume with respect to which intracranial stereotactic coordinates and trajectory are defined. The dimensions of the localizer were designed to be compatible with existing surgical planning software.

We modified and adapted a stereotactic targeting device recently developed by our group for placement of EVD catheters (Fig. 1E–G)^18^. The work envelope of this device includes the common intracranial targets of EVD (e.g., FOM, frontal ventricular horns). Therefore, its basic design was retained with 5 degrees of freedom (Fig. 1F, G): three linear (X, Y and Z axis) and two angular (arc and collar angles). The arc carrier platform (labeled with a white asterisk in Fig. 1E) was optimized for EVD catheter placement and a set of guides and reducing tubes (Fig. 1H) were developed for catheters of different sizes.

### Mechanical and imaging accuracy tests

A mechanical accuracy test fixture comprised of 5 cylinders with conical tips and a tower for attachment of the EVD key was fabricated in aluminum (Fig. 2A). The coordinates of the conical tips were known with an accuracy of 0.025 mm and referred to as the ground truth. These 5 tips provided a wide coverage of the work-envelope of the system. Each of these tips was targeted using a 150 mm long stainless-steel probe (Fig. 2A, A.1) and the coordinates were obtained by three independent observers. The arc and collar angles were kept constant at 90° and 70°, respectively. For each of the five points the difference between the ground truth and the targeting device readouts was obtained (n = 3). This difference was used to compute the 3D Euclidean error and defined as the mechanical accuracy of the device.

An imaging phantom with 35 small circular targets of known coordinates was 3D printed for imaging accuracy testing (Fig. 2C, C.1). The EVD key and localizer were mounted on the phantom and imaged in a CT scanner (Dual source Somatom Definition, Siemens AG, Munich, Germany). The images were transferred to a surgical planning computer. Fiducial registration was performed using the surgical planning software COMPASS to generate stereotactic image space. However, this step can also be performed on most other commercially available stereotactic surgical software utilizing the N-bar coordinate system. Five targets that provide a wide coverage of the work envelope of the system were chosen. They were targeted in the stereotactic image space by three independent observers to obtain their imaging coordinates (Fig. 2E). The same points were then targeted in physical space using the stainless-steel probe to obtain their physical coordinates (Fig. 2C). The 3D distance between the imaging and the physical coordinates of these targeted points were used to compute the 3D Euclidean error and defined as the imaging accuracy of the system.

### Cadaveric preclinical study

This study was deemed exempt by the Mayo Clinic Institutional Review Board. The cadavers were donated to the Mayo Clinic by patients and/or their legal guardians for the purpose of medical education and research. All experiments were carried out in accordance with institutional guidelines and regulations. A total of 10 EVD implantations were performed on two adult male human cadaver head specimens (Fig. 3A). 5 of these implantations were performed in specimen with radio-opaque vascular contrast to mimic CT angiography (CTA). The experiments were designed to closely emulate the current surgical workflow (Fig. 3). A 4.2 mm diameter metal catheter was used in place of a rubber catheter to reduce error resulting from friction-induced rumpling of the rubber in the formalin-hardened cadaver brain (Figure S1).

The EVD key and localizer were secured on the back half of the cadaver head with two midline pins and two side screws (Figs. 3B, 1C). The anterior pin was placed 17–18 cm behind the nasion in the midline. The specimen was imaged in a CT scanner with a slice thickness of 0.6 mm (Fig. 3C). The images were transferred to a surgical planning computer. COMPASS software was used for fiducial registration and surgical planning. A target in the frontal horn of lateral ventricles (Fig. 3D) and a trajectory which avoids the eloquent cortex as well as CT visible vasculature were chosen. The software provided the coordinates for the target, X, Y and Z value, as well as the trajectory, arc and collar angles.

The X, Z, arc, and collar values of the surgical target were dialed into the stereotactic device (Fig. 3E) and it was locked on the EVD key at the designated Y coordinate. The site for incision was marked (Fig. 3F) followed by scalp incision and stereotactic drilling of a 5 mm diameter burr hole (Fig. 3G). The dura was punctured with a sharp needle. A metal catheter was then advanced to the target and secured in place (Fig. 3H). A post-procedure CT scan was performed to verify the catheter coordinates and trajectory using COMPASS. To account for errors involved in the frame placement and registration and in having different head shapes, all surgical steps right from the EVD key and localizer placement to the post procedure CT scans were repeated multiple times and on multiple cadaver heads. All steps were performed by a post-doctoral fellow who completed medical school training and has prior experience in cadaveric stereotactic procedures.

The first attempt success rate, targeting error (3D Euclidean error), trajectory error, catheter tip location from FOM, number of blood vessels in contact with the catheter (radiographic assessment), and surgical time were assessed and documented. The location of catheter tip on the post-procedure CT scan was evaluated as per the clinical grading systems given by Kakarla et al.^1^ and Fargen et al.^4^.

### Mathematics of the system

The 3D Euclidean error was defined as the 3D vector error between the planned coordinates/ground truth and the actual observed coordinates for the catheter/probe tip in all cases. It was calculated as follows:

$$3\hbox{D Euclidean error }= \sqrt{{({X}_{o}-{X}_{p})}^{2} + {({Y}_{o}-{Y}_{p})}^{2} + {({Z}_{o}-{Z}_{p})}^{2}}$$

where X_o_, Y_o_, and Z_o_ denote the observed values and X_p_, Y_p_, and Z_p_ denote the planned value or ground truths. The trajectory error refers to the radial deviation of the catheter from its planned trajectory in the axial (X–Y) plane of the planned target and was calculated as follows:

$$\hbox{Trajectory error }= \sqrt{{({X}_{t}-{X}_{p})}^{2} + {({Y}_{t}-{Y}_{p})}^{2}}$$

where X_t_ and Y_t_ stands for the observed X and Y coordinates of the catheter in the X–Y plane of planned target and X_p_ and Y_p_ stands for the planned values. A schematic of the 3D Euclidean and the trajectory error is provided in Fig. 3I. All data are presented as mean ± standard deviation (SD) values.

## Supplementary information

Supplementary Information.
