# Supplementary material for: Development and validation of a rapidly deployable CT-guided stereotactic system for external ventricular drainage: preclinical study
Source: Sci Rep. 2021 Sep 1;11:17492. doi: 10.1038/s41598-021-97080-2 (PMC8410845; doi:10.1038/s41598-021-97080-2)
Supplement: Supplementary file 1 — Supplementary Information. [file 41598_2021_97080_MOESM1_ESM.pdf]

Development and Validation of a Rapidly Deployable CT-Guided Stereotactic System for External Ventricular Drainage: Preclinical Study

Authors:

Abhijeet S. Barath[1,2]; Aaron E. Rusheen [1, 3]; Juan M. Rojas Cabrera[1]; Hojin Shin [1]; Charles D. Blaha[1]; Kevin E. Bennet[1,4,6]; Stephan J. Goerss[6]; Kendall H. Lee[1,5*]; Yoonbae Oh[1,5*]

Affiliation:

[1]Department of Neurologic Surgery, Mayo Clinic, Rochester, MN 55905, USA
[2]Mayo Clinic Graduate School of Biomedical Sciences, Mayo Clinic, Rochester, MN 55905, USA
[3]Medical Scientist Training Program, Mayo Clinic, Rochester, MN 55905, USA
[4]Division of Engineering, Mayo Clinic, Rochester, MN 55905, USA
[5]Department of Biomedical Engineering, Mayo Clinic, Rochester, MN 55905, USA
[6]NaviNetics Inc., Rochester, MN, 55905, USA

[*]Correspondence: Kendall H. Lee, lee.kendall@mayo.edu , Yoonbae Oh, oh.yoonbae@mayo.edu

SUPPLEMENTARY INFORMATION

Supplementary Tables

	**Catheter tip location**	**Accuracy**	**Number of catheters in each category [Number (%)]**
1	Ipsilateral frontal horn, including tip of third ventricle	Optimal/adequate	10 (100%)
2	Contralateral frontal horn or lateral ventricle/ corpus callosum/ interhemispheric fissure	Suboptimal (shallow) in non-eloquent tissue	0 (0%)
3	Brainstem/ cerebellum/ internal capsule/ basal ganglia/ thalamus/ occipital cortex/ basal cistern	Suboptimal in eloquent tissue	0 (0%)

Table S1. Accuracy of ventricular catheter placement as per criterion given by Kakarla et al., 2008.

	Catheter tip location	Accuracy	Number of catheters in each category [Number (%)]
1	Within 5 mm of ipsilateral FOM	Ideal	9 (90%)
2	≥6 mm from FOM in ipsilateral lateral ventricle	Acceptable	1 (10%)
3	Tip in third ventricle		0 (0%)
4	Tip in contralateral ventricle	Suboptimal	0 (0%)
5	Catheter not within ventricle		0 (0%)

Table S2. Accuracy of ventricular catheter placement as per criterion given by Fargen et al., 2016.

**Boundary and recommendation**	**Rationale**	**References**
**Medial**<br><br>*At least 2 cm from midline*	-The superior sagittal sinus may be displaced from midline and be present up to 6-10 mm away from it[1].<br><br>-Blood loss is reduced when burr holes are placed at least 2 cm away from midline[2].	Reis et al., 2015<br><br>Shim et al., 2018
**Posterior**<br><br>*A least 0.5 cm anterior to coronal suture. The coronal suture is an easily palpable landmark in most cases*[3,4].	-The motor cortex is situated at a distance of 2.5-4.5 cm behind coronal suture [5–7].<br><br>-The pre-central sulcus, marking the anterior boundary of motor cortex is situated 1.8-3.7 cm behind the coronal suture. The distance is highest at midline and least at the junction of coronal suture with superior temporal line[8,9].	Rivet et al., 2004<br><br>Kendir et al., 2009<br><br>Hajjay et al., 2017<br><br>Sarmento et al., 2008<br><br>Frigeri et al., 2015
**Lateral**<br><br>*Superior temporal*	-The motor strip is closest to coronal suture at its junction with superior temporal line. Therefore, staying medial to it, reduces	Sarmento et al., 2008

line	chances of injury to motor strip[6,8].<br><br>-Avoids penetration of temporalis muscle.	Kendir et al., 2009
**Anterior**<br><br>*Hairline or junction of scalp with forehead*	-Avoids scar in cosmetically sensitive area of face.	

Table S3. Recommendations for safe zone of entry for stereotactic placement of EVD catheters.

Supplementary Figures

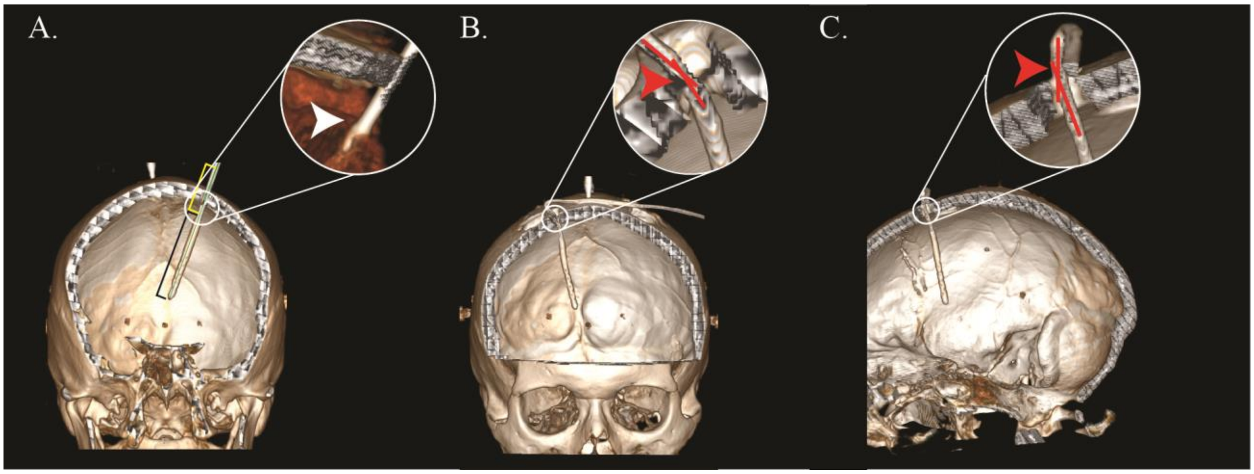

Figure S1. The pre-clinical surgery protocol was modified from clinical workflow due to practical difficulties encountered with formalin hardened cadaver heads. (**A**) 3D reconstructed CT scan showing rumpling of the rubber catheter due to friction in formalin hardened cadaver brain as shown by the black bracket. The inset shows rumpling beginning at the interface of brain tissue (shown in brown) with air. A steel catheter was therefore used for further experiments to prevent catheter distortion within the brain. (**B, C**) Coronal and sagittal reconstructed images showing deviation of catheter (see insets) at the burr hole in one of the early optimization experiments. The cause was identified as slipping of the drill bit on formalin hardened cadaver bone leading to inaccuracy in placement of stereotactic burr hole. This resulted in large deviation of catheter from its set trajectory due to mechanical restrictions by the boundaries of the burr hole. This problem was mitigated by introduction of a drill guide which is held flush with the skull surface by the surgeon and prevents the drill bit from slipping.

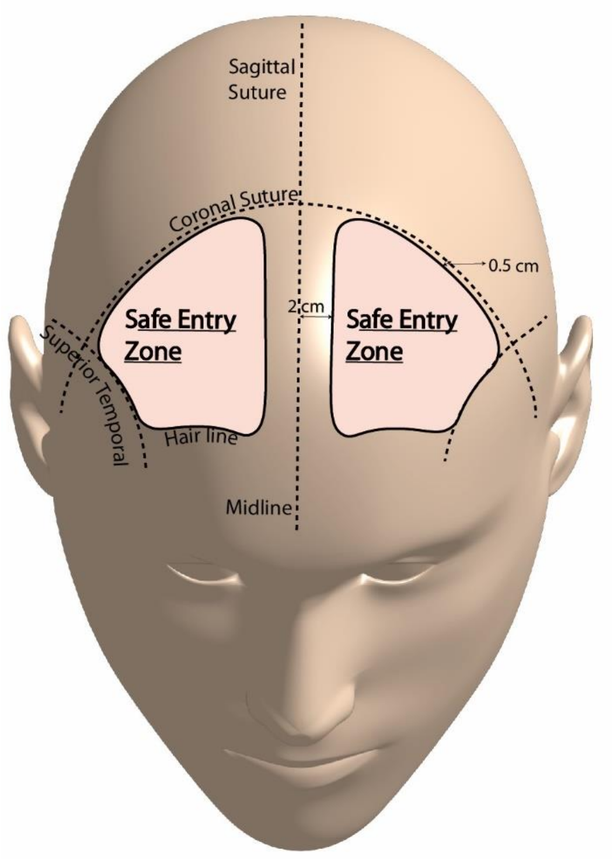

Figure S2. Boundaries of a proposed safe zone for entry for the EVD catheter to maximize patient safety. A medial boundary 2 cm lateral to midline reduces chances of injury to the superior sagittal sinus and minimizes blood loss. A posterior boundary half a cm anterior to the coronal suture reduces risk of motor cortex penetration. The lateral boundary is marked by superior temporal line below which the temporalis muscle in encountered. Lastly, the hairline of the individual is recommended as the anterior boundary to prevent scaring in cosmetically sensitive parts of the face.

References

1. Reis CVC, Gusmão SNS, Elhadi AM, et al. Midline as a landmark for the position of the superior sagittal sinus on the cranial vault: An anatomical and imaging study. *Surg Neurol Int*. 2015;6:121. doi:10.4103/2152-7806.161241

2. Shim HK, Yu SH, Kim BC, Lee JH, Choi HJ. Relationship between Clinical Outcomes and Superior Sagittal Sinus to Bone Flap Distance during Unilateral Decompressive Craniectomy in Patients with Traumatic Brain Injury: Experience at a Single Trauma Center. *Korean J neurotrauma*. 2018;14(2):99-104. doi:10.13004/kjnt.2018.14.2.99

3. Pang D, Grabb PA. Accurate placement of coronal ventricular catheter using stereotactic coordinate-guided free-hand passage. *J Neurosurg*. 1994;80(4):750-755. doi:10.3171/jns.1994.80.4.0750

4. Donovan DJ, Moquin RR, Ecklund JM. Cranial burr holes and emergency craniotomy: review of indications and technique. *Mil Med*. 2006;171(1):12-19. Accessed February 27, 2019. http://www.ncbi.nlm.nih.gov/pubmed/16532867

5. Rivet DJ, O'Brien DF, Park TS, Ojemann JG. Distance of the Motor Cortex from the Coronal Suture as a Function of Age. *Pediatr Neurosurg*. 2004;40(5):215-219. doi:10.1159/000082294

6. Kendir S, Acar HI, Comert A, Ozdemir M. Window anatomy for neurosurgical approaches: Laboratory investigation Alveolar bone loss mechanisms in leprosy View project Surgical Anatomy View project. *Artic J Neurosurg*. Published online 2009. doi:10.3171/2008.10.JNS08159

7. Hajjay AM, Mef M, Tahir AO. *Morphometric Surface Anatomy of the Pre and Postcentral Gyrus*. Vol 1.; 2017. Accessed March 15, 2019. https://www.omicsonline.org/open-access/morphometric-surface-anatomy-of-the-pre-and-postcentral-gyrus.pdf

8. Sarmento SA, Jácome DC, De Andrade EMF, Albuquerque Melo A V., De Oliveira OR, Tedeschi H. Relationship between the coronal suture and the central lobe: How important is it and how can we use it in surgical planning? *Arq Neuropsiquiatr*. 2008;66(4):868-871. doi:10.1590/S0004-282X2008000600017

9. Frigeri T, Paglioli E, de Oliveira E, Rhoton AL. Microsurgical anatomy of the central lobe. *J Neurosurg*. 2015;122(3):483-498. doi:10.3171/2014.11.JNS14315
